# Supplementary material for: Revealing the full biosphere structure and versatile metabolic functions in the deepest ocean sediment of the Challenger Deep
Source: Genome Biol. 2021 Jul 13;22:207. doi: 10.1186/s13059-021-02408-w (PMC8276468; doi:10.1186/s13059-021-02408-w)
Supplement: Supplementary file 19 — Additional file 19. Review history. [file 13059_2021_2408_MOESM19_ESM.docx]

Review History

**First round of review**

**Reviewer 1**

**Are you able to assess all statistics in the manuscript, including the appropriateness of statistical tests used? There are no statistics in the manuscript.**

**Comments to author:**

The manuscript by Chen et al describes a metagenome analysis of a surficial sediment sample obtained from the Challenger Deep at a water column depth of 10,840 m. Very few analyses have been performed on the microbiota of sediments at such a great depth, and this study represents the largest metagenomics analysis of this habitat to date.

What I liked about this paper.

In general, an impressive number of analyses were performed and the data presentation in figures and tables, including in the supplementary sections is superb.

A good amount of chemical characterization of the sediment fractions was performed.

A large amount of sequence information was garnered. This included a total of 248.65 Gb raw sequence and 6.65 Gb of assembled data. 178 metagenome assembled genomes (MAGs) were obtained with 66 more than 80% complete. One of the mags is from only the second member of a new phylum

They were able to quantitatively compare the amount of DNA associated with bacteria, archaea, microeukarya and viruses.

The functional characterization included detailed information ( Fig 2 and table S6) on the genes in MAGs associated with nitrogen, sulfur and oxygen metabolism, fermentation, utilization of aromatics, carbohydrates, hydrocarbons, hydrogen and CO oxidation. Six different CO2 fixation pathways were present. A large fraction of the MAGs included genes for autotrophy and heterotrophy. It was also interesting that a large fraction of the genomes including genes for benzoate fermentation.

The identification of hadal fungi is novel, and the presentations in Fig 3/table S7 provide superb overviews of metabolic functions.

The identification of hadal viruses is also novel, and the presentations in Fig 4/table S8/S9 of the types of viruses, virus- host relationships, and the nature of the 40 sets of viral auxiliary genes is also superb.

A large number of bacterial isolates (1070) and fungal isolates (19) were obtained and taxonomically characterized. Nineteen of the bacterial isolates could represent new species. The fungi obtained have matched sequences in the metagenome.

Where I have concerns:

The temperature of the boxcore after recovery is not presented. Thus, the potential effect of warming on the microbes present is unknown.

Only one boxcore sample was recovered. So, this entire story rests on one sample, divided into three different depth horizons. Because of this the generality of the data among nearby sediments, let alone in quantitative comparisons with more distant (e.g., in versus out of trench) samples is an unknown.

Another concern is that boxcores are notorious for disturbing sediment stratification..

No description of changes in the community structure with depth in the sediment is provided.

Throughout the portions of the paper that address the functional characteristics of the MAGs and metagenome data very little perspective is provided. For example:

-Is the distribution of aerobic/anaerobic metabolism surprising for a marine surficial sediment?

-Is the fact that 136/178 mags indicate capabilities for facultative anaerobic metabolism surprising?

-Are the sulfur metabolism genes present in the fungi surprising?

-Is it surprising that the largest category of viral auxiliary genes were for carbohydrate utilization?

So, in general the functional characterization provide no perspective on what types of metabolisms are over or under represented relative to other studies of other sediments.

None of the culturing was done in a manner that would have enriched for species adapted to grow under the physical conditions that existed in their sediment material prior to recovery. No evidence was obtained that any of the isolates are psychrophiles (although some did grow at 4C and might be psychrophiles) and no growth following incubations at 100 MPa was reported. The diversity of the microbes obtained was very low as was their presence in the metagenome data. The types of microbes cultured are most likely derived from those that descended from much higher up in the water column.

The authors performed some of their enrichments on agar media in Petri plates under anaerobic conditions. But, these plates had been first exposed to air. Why were no experiments performed to isolate strictly anerobic microbes from the sediments?

**Reviewer 2**

**Are you able to assess all statistics in the manuscript, including the appropriateness of statistical tests used?** There are no statistics in the manuscript.

**Comments to author:**

This paper presents an interesting and technically well conducted survey of microbial communities of the Challenger Deep region of the Mariana Trench using based on metagenome-assembled genomes and cultivation of member species with subsequent characterisation by 16S and ITS amplicon sequencing. Secondary analysis are based on analysis of funtional capacity, and dissecion of microeukaryote, fungal and viral members of the community. The samples are taken from three sediment depths from location at 10840m depth, with technical replicates made at the sequencing library level, and I am very pleased to see that the authors have not attempted to over-intepret the data in relation to ecosystem function which is a common flaw in papers of this kind. In total there is close to 250Gbp (211Gbp post-QC and filtering) of sequence obtained at 150bp read length using Illumina.

Comments:

1. p29. Metagenome assembly, mapping and binning. Some more detail is required here. Was single-sample or co-assembly was performed? If the former, was dereplication performed? Line 711, which cutoff was applied for each binner? In total how many bins were actually recovered aross the entire quality spectrum?

2. p10-16. This is a very long section given that is only based on describing known metabolic machinery that are encoded by the 178 MAGs. While this section is better written than many papers in this field, can the authors at least consider shortening it? Secondly, because of the high proportion of medium quality genomes, any claim of missing components can be attributed in part to genome incompleteness (due to, for example, mis-assembly or false negatives) and some mention of these limitations should included. Some mention of the fact that this paper is only deals with genes of known molecular function, and not unknown function function should be explicitly discussed e.g. see recent work on this problem (https://www.biorxiv.org/content/10.1101/2020.06.30.180448v5)

3. p9. "...illustrate the evolutionary relatiomships..." . In this, and many other papers in this field, the phylogenetic tree is simply being used as a visual scaffold to display, can you please modify this statement for improved accuracy?

4. 16S/ITS analysis. What V-regions are being targeted and how long are the amplicons? Is there any insight into whether these culture isolates recapitulate what was captured in the MAG analysis? In other words, to what extent are these a separate complement of species from the community from those captured by MAGs?

**Authors Response**

**Point-by-point responses to the reviewers’ comments:**

**Reviewer #1:** The manuscript by Chen et al describes a metagenome analysis of a surficial sediment sample obtained from the Challenger Deep at a water column depth of 10,840 m. Very few analyses have been performed on the microbiota of sediments at such a great depth, and this study represents the largest metagenomics analysis of this habitat to date.

What I liked about this paper.

In general, an impressive number of analyses were performed and the data presentation in figures and tables, including in the supplementary sections is superb.

A good amount of chemical characterization of the sediment fractions was performed.

A large amount of sequence information was garnered. This included a total of 248.65 Gb raw sequence and 6.65 Gb of assembled data. 178 metagenome assembled genomes (MAGs) were obtained with 66 more than 80% complete. One of the mags is from only the second member of a new phylum

They were able to quantitatively compare the amount of DNA associated with bacteria, archaea, microeukarya and viruses.

The functional characterization included detailed information ( Fig 2 and table S6) on the genes in MAGs associated with nitrogen, sulfur and oxygen metabolism, fermentation, utilization of aromatics, carbohydrates, hydrocarbons, hydrogen and CO oxidation. Six different CO2 fixation pathways were present. A large fraction of the MAGs included genes for autotrophy and heterotrophy. It was also interesting that a large fraction of the genomes including genes for benzoate fermentation.

The identification of hadal fungi is novel, and the presentations in Fig 3/table S7 provide superb overviews of metabolic functions.

The identification of hadal viruses is also novel, and the presentations in Fig 4/table S8/S9 of the types of viruses, virus- host relationships, and the nature of the 40 sets of viral auxiliary genes is also superb.

A large number of bacterial isolates (1070) and fungal isolates (19) were obtained and taxonomically characterized. Nineteen of the bacterial isolates could represent new species. The fungi obtained have matched sequences in the metagenome.

We thank the reviewer for the comments on our manuscript.

Specific comments and questions are addressed in the following point-by-point response.

Where I have concerns:

The temperature of the boxcore after recovery is not presented. Thus, the potential effect of warming on the microbes present is unknown.

Response：

This is one of the concerns with our boxcore, and also with most deep-sea landing instruments for sediment study. There is limited ability for the instrument to preserve the original sediment sample temperature when surfacing. (So far there is reported success for preserving water sample temperature.)

The environment temperature recorded by the box corer was 2-4 ^o^C at ocean floor of the Challenger Deep, and 27-28 ^o^C at ocean surface at the time. (The sediment core was colder than the environment temperature when surfacing, but its exact temperature was not recorded.)

Increase in temperature certainly had some effects on the sediment microbes. We took steps in hope to minimize the impact: 1) the boxcore was swiftly processed once brought to the deck; 2) the core was subsampled and stored immediately in a -80 ^o^C freezer on the ship. These would help maintain the big-picture characteristics of the microbial communities, reducing effects for study using metagenome sequencing.

We thank the reviewer for the comment that brought the issue to our attention -- we have referred to this potential issue in the revised manuscript (page 7, lines 140-142; red text).

Only one boxcore sample was recovered. So, this entire story rests on one sample, divided into three different depth horizons. Because of this the generality of the data among nearby sediments, let alone in quantitative comparisons with more distant (e.g., in versus out of trench) samples is an unknown.

Response:

Yes, we had some difficulties to get additional intact sediment cores from the deepest site (~11000 meter) in the Challenger Deep after one successful attempt. It has been technologically challenging and indeed difficult to get intact boxcore samples from the deepest ocean bottom.

Three layer samples were analyzed by dividing the boxcore at depth 0-5, 5-10, and 10-14 cm. These samples enabled us for a deep metagenomic sequencing study, which generated the first metagenomics snapshot for the microbial community in the deepest marine biosphere, offering detailed information on microbial diversity and their metabolic potentials.

We also launched with it, an extensive large-scale cultivation effort for isolation of the Challenger Deep sediment microbes, using 24 different type media with various culture conditions (Additional file 1: Table S10).

To alleviate concerns about generality, we performed some broad comparative analyses with: 1) data of other Mariana Trench (MT) sediment samples (they were found to be exclusively based on 16S rRNA sequencing); and 2) data of deep-sea sediment samples from non-MT sources that are based on metagenomic sequencing.

1) We found four previous studies on MT sediment microbial communities [1-4], all of which were based on 16S rRNA sequencing (summarized in the attached “Addition file 1: Table S14”). Our results agree with the previous studies, in which Proteobacteria, Chloroflexi, Actinobacteria, Planctomycetes, and Firmicutes were the most abundant bacteria, and Thaumarchaeota and Woesearchaeota the most abundant archaea in the MT sediment habitats. On the other hand, large variations of microbial abundance were observed among microbial communities in different MT sites. For example, Thaumarchaeota was the most abundant phylum ranging from 0.67% to 67% in one study [4] and from 0.5% to 40% in another [2], whereas it accounted for ~5% in our metagenomics analysis, although Thaumarchaeota was the top archaea in all the cases. The different technical approaches may contribute to the variations, in addition to many environmental factors in these habitats. Our deep metagenomics study offered more details, and hence became metagenomics reference on the microbial diversity and metabolic functions of the MT sediment microbiome.
2) We obtained two relevant sets of metagenomics sequencing data from deep-sea sediment samples of non-MT sources, i.e. the Yap Trench (YT) (abyssal sites: 4435m, and 4568m; and hadal site: 6578m) [5] and the New Britain Trench (NBT) (8720 meter) (unpublished). Taxonomic classification were run on them using the same procedure as described in our manuscript. The side-by-side comparison of the results with the same pipeline convincingly reveals the characteristics for each hadal sediment microbiome (summarized in the attached “Addition file 1: Table S15”). Proteobacteria was the most abundant bacteria in all three sediment samples, whereas Chloroflexi, Actinobacteria, Planctomycetes, and Gemmatimonadetes were significantly lower in the Yap Trench sediments than those of the other two. Thaumarchaeota proves to be the highest in the YT sediment, but much lower in that of MT and NBT. In an overall perspective, our study illustrated the distinct community profiles in the Challenger Deep sediment habitat, offering a valuable reference for the microbial diversity and metabolic functions of the hadal sediment microbiome.

We appreciate the reviewer’s comments, and have added two Supplementary Tables (Table S14 and Table S15) and supplemented related discussion (page 8, lines 164-173; page 11, 239-240; red text) in the revised manuscript .

Another concern is that boxcores are notorious for disturbing sediment stratification..

Response:

We appreciate the reviewer’s comment. This is another one of the concerns with the sampling in our study and also in others’ using boxcores.

To alleviate it, we made sure to use the core that was intact when we retrieved. The box corer was sealed at the seafloor as described (refer to page 28: “Sample collection”), and the sediment stratification was preserved during surfacing. (Note those with leaked/ incomplete core were not used for sequence study.)

Also the size of our box corer was fairly large [20 cm x 20 cm x 25 cm (height)] (page 28) which helped keeping stable the sample structure.

While our study was designed to analyze the vertical variations in microbial communities and associated metabolic functions, we were mindful of this possible issue and did not over-interpret the results -- we have added reference to this potential issue in the revised manuscript (page 28, lines 669-670; red text).

No description of changes in the community structure with depth in the sediment is provided.

Response:

We appreciate the reviewer’s comment. We described, in general, that the overall relative abundance of archaea in the microbiome decreased with sediment depth - similar to findings in a previous study [3] (page 8, line 141). However, the relative abundance of both the microeukaryotes and marine viruses increased with sediment depth.

Overall bacteria are the most abundant microbe, accounting for 82.07% of the community sequence. We found many had consistent abundance between MT-1 and MT-2, but became significantly different in MT-3 (Fig. 1A). For example, Alphaproteobacteria, Deltaproteobacteria, Betaproteobacteria, Chloroflexi, lanctomycetes, Acidobacteria, Cyanobacteria, Verrucomicrobia, and Euryarchaeota had higher levels in MT-1 and MT-2, but were reduced in MT-3. However, the reverse was true for Gammaproteobacteria, Fusobacteria, and Unclassify, which became significantly higher in MT-3.

The 10-cm line appeared to be a boundary that separated environments with different community compositions. It was suggested that oxygen penetration being limited at this depth could be a contribution factor [6].

However, we were also cautious not to over-interpret the results (related to the reviewer’s last comment regarding sediment stratification).

We have added description of changes with depth in the community structure in the revised manuscript (page 8, lines 171-173, 176-178; red text).

Throughout the portions of the paper that address the functional characteristics of the MAGs and metagenome data very little perspective is provided. For example:

-Is the distribution of aerobic/anaerobic metabolism surprising for a marine surficial sediment?

-Is the fact that 136/178 mags indicate capabilities for facultative anaerobic metabolism surprising?

-Are the sulfur metabolism genes present in the fungi surprising?

-Is it surprising that the largest category of viral auxiliary genes were for carbohydrate utilization?

So, in general the functional characterization provide no perspective on what types of metabolisms are over or under represented relative to other studies of other sediments.

Response:

We appreciate the reviewer’s comments ~ we have addressed them in a broader perspective as described in the followings.

-Is the distribution of aerobic/anaerobic metabolism surprising for a marine surficial sediment?

-Is the fact that 136/178 mags indicate capabilities for facultative anaerobic metabolism surprising?

In a broader perspective, the marine surficial sediments resided near the water and mud interface, for which critical questions have been raised about the metabolic activities and geochemical process at this juncture. It is believed that the oxygen contents in surficial sediments from the Challenger Deep have a major effect on the compositions of microbial communities and their metabolism.

-- This is the reason why we preformed analysis from the angle of aerobic/anaerobic metabolism for the hadal sediment microbes in the first place. (Note: it has not been done from this angle for hadal sediments, nor has it been done for other deep-sea sediments).

From our MAG analysis, we found both the aerobic respiratory and anaerobic respiration pathways were equally widely-distributed (87% vs 81%) in the Challenger Deep sediment microbiome. In comparison, in sediment samples from deep petroleum seeps (2800-3200 m) in the Eastern Gulf of Mexico, a distinctive profile was observed: anaerobic respiration accounting for 98% MAGs and aerobic respiration for 25% (a total of 82 MAGs with >50% completeness and <10% contamination) [7]. This microbial community was enriched for anaerobic respiration metabolism, apparently driven by the local presence of hydrocarbons like alkanes, aromatic compounds, etc.

On the other hand, in the Guaymas Basin hydrothermal sediments that were organic-rich with hydrocarbons, and where oxygen levels were depleted within the depth of few millimeters, considerably more MAGs were positive for anaerobic respiration metabolism (99.46% MAGs ) than for aerobic respiration (26.68% MAGs) [8]. These results suggested a similar mechanism (driven by the presence of hydrocarbons and depleted oxygen) was at work for the Guaymas Basin hydrothermal sediment habitat.

Regarding the facultative anaerobic metabolism, ~76% MAGs from the Challenger Deep sediment microbiome were positive, pointing to a high proportion that reflects the microbial metabolic versatility and ability to adapt to endemic habitats or environmental disturbance.

In contrast, the sediment from the deep petroleum seeps in the Eastern Gulf of Mexico had 25% MAGs capable of facultative anaerobic metabolism [7], whereas the Guaymas Basin hydrothermal sediments had 26.68% MAGs capable of it [8] . The microbial communities appeared adapted to each particular habitat, but were restricted in their ability to adjust their metabolism when transferred to aerobic conditions.

We appreciate the reviewer’s comments regarding their perspective, and have supplemented related discussions in the revised manuscript (pages 14-15, lines 326-330, 334-335; red text).

-Are the sulfur metabolism genes present in the fungi surprising?

Response:

It has long been known that sulfur metabolism has a special role in environment microbial metabolism and in driving geochemical processes. Thus when we found the hadal fungi from the Challenger Deep sediment possessed the complete pathways for assimilatory sulfate reduction, dissimilatory sulfate reduction, and sulfide oxidation, we realized immediately that the hadal fungi -- like hadal bacteria, could take part in the sedimentary sulfur cycle.

Although the sulfur metabolism pathways were previously known to exist in many fungi, we have not seen them in reported studies on marine sediment fungal community, probably because they mostly used ITS for investigating fungal community, which lacked the means for diving into gene-function details.

Thus, by metagenomically characterizing the fungal community in the Challenger Deep sediment for the first time, we presented evidence that supported the roles of fungi in sulfur cycling. (An important question is also raised on how large the role the fungal community played in geochemical flux and balance.) .

Previous studies reported that fungi are able to oxidase sulfur and sulfur compounds and release sulfate to environment [9,10]. Thus, fungi could provide sulfate to sulfate-reducing bacteria (SRB) and could potentially be involved in the sulfur cycle in subsurface environment [11]. Interestingly, we also found SRB, e.g. Desulfobacterales and Desulfuromonadales (within the class Deltaproteobacteria), and Nitrospirae (phylum) in the Challenger Deep sediment (Fig. 1). It corroborates the evidence for the existence of sulfide oxidation reactions and the roles that fungi that play in sulfur cycling in the hadal sediment habitat.

We appreciate the reviewer’s comments, and have supplemented related discussion in the revised manuscript (page 19, lines 453-455; page 21, lines 485-491; red text).

-Is it surprising that the largest category of viral auxiliary genes were for carbohydrate utilization?

Response:

Our study provided a snapshot on the hadal sediment viral community and their perspective hosts, implying their possible roles in facilitating hosts’ metabolism in the deepest marine habitat.

Further, our data added to the details that the Challenger Deep sediment viruses were most involved in carbohydrate degradation reactions -- one of the largest AMG categories. Specifically, 45 carbohydrate metabolism-related genes were identified by searching against the CAZymes database. They included 31 GHs (glycosidases or glycosyl hydrolases), six PLs (polysaccharide lyases), five GTs (glycosyltransferases), two CBM (carbohydrate binding modules), and one AA (auxiliary activities).

The results provided the first evidence the Challenger Deep sediment viruses may heavily manipulate carbohydrate metabolism of hosts, especially promoting carbohydrate degradation in hosts in an oligotrophic habitat.

Since no metagenomic data were reported for hadal sediment viruses, we turned to other marine viral communities for comparison.

1) The marine sediment viral communities in a cold seeps (ranging from 800 to 3200 m below the sea level) were recently reported [12]. AMGs for carbohydrate metabolism are among the top AMG groups, including 32 carbohydrate-active enzymes: six GHs, seven PLs, 14 CBM, and five CE (Carbohydrate Esterases). However, differently from the Challenger Deep sediment viruses, they were concentrated on functions of CBM and CE.
2) In the viral communities in the deep-sea sediments of Southwest Indian Ocean (1676 to 3493 m below the sea level) [13], AMGs for carbohydrate metabolism were the second largest group. They were found to be mainly involved in pentose phosphate pathway, glyoxylate and dicarboxylate metabolism, citrate cycle, etc. Their roles were concentrated in facilitating the survival of the host cells in the nutrient-limiting habitat.
3) We lastly compared with the Tara Oceans Viromes (TOV) dataset that are largely composed of epipelagic and mesopelagic viral populations [14].

Although AMGs for carbohydrate metabolism were also found to be the largest group for TOV, they concentrated on pathways different from our hadal sediment virome. They were mainly involved in galactose metabolism (48.56%), Glycosyltransferases (GTs, 11.66%), capsular polysaccharide biosynthesis /D-mannose metabolism (5.00%), pentose phosphate pathway (6.79%), glycoside hydrolase family 73 (GHs, 3.03%), fructose and mannose metabolism (3.00%) and dehydrogenase (1.88%). The high proportion of AMGs for galactose metabolism in TOV was not discussed [14]. However, we suspect that they may facilitate the hosts’ (e.g. Cyanobacteria and Alphaproteobacteria) metabolism in the epipelagic and mesopelagic habitats rich in galactose that was generated by macro algae through fermentation [15].

Taken together, the distinct profile of AMGs for carbohydrate utilization in our Challenger Deep sediment virom reflects the differentiation of viral niche to microbial hosts living in the hadal habitat of high-hydrostatic pressure and oligotrophy.

(We noted in our manuscript (pages 22-23): the most frequent viral hosts were Cyanobacteria and Alphaproteobacteria in epipelagic and mesopelagic ocean waters, whereas the most frequent hosts in the Challenger Deep sediment were Firmicutes (mainly Bacilli) and Bacteroidetes, followed by Euryarchaeota (including uncultured marine group II/III and Diaforarchaea), unclassified Chloroflexi, and Alphaproteobacteria.)

We appreciate the reviewer’s comments, and have supplemented related discussion on viral auxiliary genes for carbohydrate utilization in the revised manuscript (page 23-24, lines 556-563; red text).

None of the culturing was done in a manner that would have enriched for species adapted to grow under the physical conditions that existed in their sediment material prior to recovery. No evidence was obtained that any of the isolates are psychrophiles (although some did grow at 4C and might be psychrophiles) and no growth following incubations at 100 MPa was reported. The diversity of the microbes obtained was very low as was their presence in the metagenome data. The types of microbes cultured are most likely derived from those that descended from much higher up in the water column.

Response:

By design we sought to characterize the Challenger Deep sediment microbial community by two different means: metagenomics and large-scale culture. The large-scale culture would be the most extensive effort so far for isolation of the hadal sediment microbes using 24 different type media with various culture conditions (Additional file 1: Table S10).

Because the scale of the culture made it difficult (if not impossible) to place them under pressured conditions (which would serve as an enrichment mechanism), we chose to apply pressured culture for colonies after they emerged from the first-step large-scale culture.

The isolated colonies (over 2000) were found to belong to 4 phyla, 7 classes, 18 orders, 25 families, and 40 genera. They were highly redundant in three genera: Halomonas, Pseudoalteromonas, and Idiomarina. In addition, 19 bacteria isolates were candidate for new species of five different classes.

Although they accounted for a relatively small diversity in contrast to the microbial presence in the Challenger Deep metagenome, our results represented the broadest isolates taxonomy-wise from the hadal sediment. Notably, most of the rest microbes present in the metagenome have not been and probably can not be (solely) cultured.

We selected representative bacteria (including Halomonas, Pseudoalteromonas, and Idiomarina) and fungi from ours isolates and placed them under pressured culture (100 MPa). Based on the results, they were likely piezotolerant microbes: they remained structurally intact; they showed signs of division and stressed under elevated pressure, as sister cells were connected after cell divisions under elevated pressure; and they can restore rapid growth when re-plated in normal conditions. The evidence supported them for being piezotolerant, but not for being piezophiles or psychrophiles, for which we were careful not to make an over-claim.

The results raised an important question (also brought up by the reviewer) about the source of these bacterial isolates. We agreed with the possibility that they may be derived from the microbes that descended from the water column.

Supported by the metagenomics data and our bacterial isolates, it is conceivable that a substantial portion of the microbiome in the Challenger Deep sediment is composed of, or constantly furnished with microbes descending from the water column.

Then the big question is how much does this ‘descending’ process contribute to the diversity and metabolic functions of the hadal sediment microbial community? It is puzzling that the metagenomics results did not show a significant presence of the known extremophiles that are supposed to be adapted to proliferate in the hadal sediment habitat. Both the metagenomics data and the large-scale culture results may suggest a significant role of the ‘descending’ process for constitution of microbial community in the deepest marine biosphere.

We appreciate the reviewer’s comments, and have supplemented related discussion in the revised manuscript (pages 25-26, lines 602-603, 605-610; red text).

The authors performed some of their enrichments on agar media in Petri plates under anaerobic conditions. But, these plates had been first exposed to air. Why were no experiments performed to isolate strictly anerobic microbes from the sediments?

Response:

The hadal sediment cores were exposed to air when they were retrieved from box corer after surfacing on the sea. Although we made best efforts (see below) to reduce their exposure to oxygen, it may not be feasible for culture of strictly anaerobic microbes. Our effort using expanded culture conditions is likely to favor facultative anaerobic bacteria.

We hope to clarify on the anaerobic procedure we used:

1) The sediment core was immediately sub-sampled. The sediment samples were sealed in air-tight bags with residue air removed, before they were stored at 4 ^o^C.

2) For anaerobic culture, the agar media plates were first treated with AnaeroPack (Mitsubishi Gas Chemical Company, Inc., Tokyo) to remove oxygen before they were used for anaerobic culture.

We appreciate the reviewer’s comments, and have made clarifications on the details of anaerobic procedure in the revised manuscript (pages 34-35, lines 828-830, 835-837; red text).

**Reviewer #2:** This paper presents an interesting and technically well conducted survey of microbial communities of the Challenger Deep region of the Mariana Trench using based on metagenome-assembled genomes and cultivation of member species with subsequent characterisation by 16S and ITS amplicon sequencing. Secondary analysis are based on analysis of funtional capacity, and dissecion of microeukaryote, fungal and viral members of the community. The samples are taken from three sediment depths from location at 10840m depth, with technical replicates made at the sequencing library level, and I am very pleased to see that the authors have not attempted to over-intepret the data in relation to ecosystem function which is a common flaw in papers of this kind. In total there is close to 250Gbp (211Gbp post-QC and filtering) of sequence obtained at 150bp read length using Illumina.

We thank the reviewer for the comments on our manuscript.

Specific comments and questions are addressed in the following point-by-point response.

Comments:

1. p29. Metagenome assembly, mapping and binning. Some more detail is required here. Was single-sample or co-assembly was performed? If the former, was dereplication performed? Line 711, which cutoff was applied for each binner? In total how many bins were actually recovered aross the entire quality spectrum?

Response:

We thank the reviewer for the comments.

Co-assembly was performed using sequencing data from the three layers, MT-1, MT-2, and MT-3. (Note for the co-assembly approach, we also removed the duplicated reads likely due to PCR performed for building Illumina sequencing libraries before assembly was carried out.)

We have updated the manuscript clarifying the details (page 7, lines 144-145; red text)

For the two binners, the length cutoff for CONCOCT is 1 kb, and for Metabat 1.5 kb. 310 bins were recovered from CONCOCT, and 400 bins from Metabat. After merging, a total of 418 bins were recovered across the entire quality spectrum.

(Note a further filtering with cutoff of 50% completeness and 10% contamination resulted in 178 draft genomes (MAGs) that were above the median quality standards.)

We have added details clarifying the parameters and cutoff used in assembly and binning process in the revised manuscript (page 9, lines 184, 185, 187; page 30, lines 728-729, 732-733; red text).

2. p10-16. This is a very long section given that is only based on describing known metabolic machinery that are encoded by the 178 MAGs. While this section is better written than many papers in this field, can the authors at least consider shortening it? Secondly, because of the high proportion of medium quality genomes, any claim of missing components can be attributed in part to genome incompleteness (due to, for example, mis-assembly or false negatives) and some mention of these limitations should included. Some mention of the fact that this paper is only deals with genes of known molecular function, and not unknown function function should be explicitly discussed e.g. see recent work on this problem (<https://www.biorxiv.org/content/10.1101/2020.06.30.180448v5>)

Response：

We thank the reviewer for the two suggestions.

Regarding the metabolic functions encoded by the 178 MAGs, our discussion was written focusing on three different angles that have not been previously addressed, especially for the MT hadal sediment.

1) Heterotrophy vs. Autotrophy: we observed a significant taxonomic expansion of mixotrophic microbes, indicating an adaptive niche that mixotrophy confers to microbes living in an oligotrophic habitat like the Challenger Deep sediment.

We have shorted the section “ inorganic carbon fixation” (pages 11, 12).

2) Aerobic vs Anaerobic Respiration: The high proportion of facultative anaerobes in the MT sediment reflects the microbial metabolic versatility and the ability to adapt by the hadal microbes to endemic habitats or environmental disturbance.

We have shorted it by moving the section “potential for different types of fermentation that degrade organic matters” (page 14; lines 323-325) to “Additional file 3 Analysis of fermentation”.

3) The important aspects of chemolithotrophy were discussed for the MT sediments. No changes were made since this section was very compact.

Regarding to the limitation of our results in dealing with genome incompleteness, and genes of known and unknown functions, we agree that it is an extremely important question to address in order to have a thorough understanding of the MT hadal sediment microbiome.

We have updated the manuscript with pointed discussions in following two aspects (page 11, lines 241-244; red text).

1) Though MAGs analysis, we must keep in mind that many metabolic pathways are uncomplete, for which most missing components can be attributed in part to imcomplete assembly.

2) Special attention needs to be paid to the large quantity of assembled sequences with unknown function. It is necessary to approach this significant gap by develop new means to bridge the known-unknown gap in metagenomic research [16].

3. p9. "...illustrate the evolutionary relatiomships..." . In this, and many other papers in this field, the phylogenetic tree is simply being used as a visual scaffold to display, can you please modify this statement for improved accuracy?

Response:

We have made modifications to the statement: “To illustrate the taxonomic diversity of the hadal sediment microbes” (page 9, line 206, red text).

We thank the reviewer for the comment.

4. 16S/ITS analysis. What V-regions are being targeted and how long are the amplicons? Is there any insight into whether these culture isolates recapitulate what was captured in the MAG analysis? In other words, to what extent are these a separate complement of species from the community from those captured by MAGs?

Response:

We apologize for the incomplete details regarding 16S/ITS analysis.

For analysis of 16S for isolated bacterial strains, two primer sets: 8F/805R and 8F/1492R, were employed. 8F/805R targeted V1-V4 regions and 8F/1492R covered V1-V9 regions, which generated the PCR-amplified tags with length of 805 and 1492 bp, respectively.

For fungi, the primer set, ITS-F/ITS-R was employed to amplify ITS sequence, which generated the PCR-amplified tag with length of 578 bp.

We have added them in the revised manuscript (pages 35, lines 847-850, 853-854; red text).

We gained much insight (from the culture isolates) into the microbiome of the MT hadal sediment. While being the most extensive large-scale culture of microbial isolates from the Challenger Deep hadal sediment, our work exemplified the small portion of culturable microbes in contrast to the community background illustrated by MAG analysis.

1) In details, although over 2000 colonies were isolated, they concentrated to four phyla (Proteobacteria, Bacteroidetes, Actinobacteria, and Firmicutes) out of more than 20 phyla or candidate phyla represented by the MAGs. Our results indicated the culturabe isolates were limited, despite that we used 24 different media and combined them with various culture conditions (Table S10).
2) However, MAG analysis gives sometimes sparse information. For example, many of the MAG can only be classified to phylum level. Isolated microbes greatly complemented the lack of taxonomy details from MAG analysis. For example, among the 7 classes represented by isolated microbes, only four of them were definitely defined by MAG analysis. Furthermore, nineteen bacterial isolates from five classes were found to be candidate for new species (Table S13).

So on complementing MAG analysis, culture isolates gave more detailed snap shots on the culturable section of microbial community and providing precious materials for physiological and mechanical study of piezotolerant microbes.

We have added discussions on the ‘complement’ aspect of isolated microbes in the revised manuscript (page 25, lines 602-605; red text). We thank the reviewer for the comments.

Reference

1.	Peoples LM, Grammatopoulou E, Pombrol M, Xu X, Osuntokun O, Blanton JM, Allen EE, Nunnally CC, Drazen J, Mayor DJ: Microbial Community Diversity within Sediments from Two Geographically Separated Hadal Trenches. Frontiers in Microbiology 2019, 10:347.

2.	Cui G, Li J, Gao Z, Wang Y: Spatial variations of microbial communities in abyssal and hadal sediments across the Challenger Deep. PeerJ 2019, 7:e6961.

3.	Nunoura T, Nishizawa M, Hirai M, Shimamura S, Harnvoravongchai P, Koide O, Morono Y, Fukui T, Inagaki F, Miyazaki J: Microbial Diversity in Sediments from the Bottom of the Challenger Deep, the Mariana Trench. Microbes and Environments 2018, 33:186-194.

4.	Hiraoka S, Hirai M, Matsui Y, Makabe A, Minegishi H, Tsuda M, Juliarni, Rastelli E, Danovaro R, Corinaldesi C, et al: Microbial community and geochemical analyses of trans-trench sediments for understanding the roles of hadal environments. ISME J 2020, 14:740-756.

5.	Xinxu, Zhang, Wei, Xu, Yang, Liu, Mingwei, Cai, Zhuhua, Luo: Metagenomics Reveals Microbial Diversity and Metabolic Potentials of Seawater and Surface Sediment From a Hadal Biosphere at the Yap Trench. Frontiers in Microbiology 2018.

6.	Wenzhöfer F, Oguri K, Middelboe M, Turnewitsch R, Toyofuku T, Kitazato H, Glud RN: Benthic carbon mineralization in hadal trenches: Assessment by in situ O2 microprofile measurements. Deep Sea Research Part I: Oceanographic Research Papers 2016.

7.	Dong X, Greening C, Rattray JE, Chakraborty A, Chuvochina M, Mayumi D, Dolfing J, Li C, Brooks JM, Bernard BB: Metabolic potential of uncultured bacteria and archaea associated with petroleum seepage in deep-sea sediments. Nature communications 2019, 10:1-12.

8.	Dombrowski N, Teske AP, Baker BJ: Expansive microbial metabolic versatility and biodiversity in dynamic Guaymas Basin hydrothermal sediments. Nature communications 2018, 9:4999.

9.	Sterflinger, Katja: Fungi as Geologic Agents. Geomicrobiology Journal 2000, 17:97-124.

10.	Wainwright M, Grayston SJ: Accumulation and oxidation of metal sulphides by fungi. Special Publications of the Society for General Microbiology 1989.

11.	Sohlberg E, Bomberg M, Miettinen H, Nyyssönen M, Salavirta H, Vikman M, Itävaara M: Revealing the unexplored fungal communities in deep groundwater of crystalline bedrock fracture zones in Olkiluoto, Finland. Frontiers in Microbiology 2015, 6.

12.	Li Z, Pan D, Wei G, Pi W, Dong X: Deep sea sediments associated with cold seeps are a subsurface reservoir of viral diversity. The ISME Journal 2021.

13.	Extraordinary diversity of viruses in deep‐sea sediments as revealed by metagenomics without prior virion separation. Environmental Microbiology 2020.

14.	Roux S, Brum JR, Dutilh BE, Sunagawa S, Duhaime MB, Loy A, Poulos BT, Solonenko N, Lara E, Poulain J, et al: Ecogenomics and potential biogeochemical impacts of globally abundant ocean viruses. Nature 2016, 537:689-693.

15.	Wei N, Quarterman J, Jin YS: Marine macroalgae: an untapped resource for producing fuels and chemicals. Trends in Biotechnology 2013, 31:70-77.

16.	Vanni C, Schechter MS, Acinas SG, Barberán A, Buttigieg PL, Casamayor EO, Delmont TO, Duarte CM, Eren AM, Finn RD, et al: Unifying the known and unknown microbial coding sequence space. bioRxiv 2021:2020.2006.2030.180448.

**Second round of review**

**Reviewer 1**

I appreciate the author's consideration of reviewer recommendations. The points that remain are as follows:

Genome Biology re-review

The materials and methods should indicate that the temperature of the recovered core was not obtained, but the surface water was 27-28C, and the temperature at the ocean floor was 2-4C.

Line 659 - sample collection. The section needs to be rewritten to make it clear to readers that only one sediment cores was obtained.

Line 660 - The one sediment sample obtained…

Line 662 - The sediment core was…

Line 669 - Only the one sediment core with preserved sediment stratification was used for this study.

Line 334. Change to “Compared to the microbial communities…”

Line 607. Why do you think the descending (culturable) microbes might have a significant role in the hadal sediment microbiome when they were a tiny fraction of the overall community abundance, as reflected in the metagenomes? The authors should quantify the fraction of microbiome represented by the cultured isolates.

Line 835. Anaerobic agar culturing. Despite the treatment of the agar to remove oxygen, the agar media were inoculated under aerobic conditions in the lab correct? A anaerobic hood was not used correct? This information should be included. And the authors should make it clear that the conditions used would not have enabled the isolation of strict anaerobes, only facultative anaerobes.

**Reviewer 2**

No further comments, the authors have addressed my concerns and the paper is much improved.

**Authors Response**

**Point-by-point responses to the reviewers’ comments:**

**Reviewer 1**

I appreciate the author's consideration of reviewer recommendations. The points that remain are as follows:

Genome Biology re-review

The materials and methods should indicate that the temperature of the recovered core was not obtained, but the surface water was 27-28C, and the temperature at the ocean floor was 2-4C.

Per the reviewer’s comment, we added the temperature information in the section 'Materials and methods' in the revised manuscript (page 28, lines 14-16, red text).

Line 659 - sample collection. The section needs to be rewritten to make it clear to readers that only one sediment cores was obtained.

We have rewritten the texts to clarify that one intact sediment core was used in this study in the paragraph "Sample collection and geochemical measurements" (page 28, lines 1-11; red text). For details, please refer to the next comments

Line 660 - The one sediment sample obtained…

Line 662 - The sediment core was…

Line 669 - Only the one sediment core with preserved sediment stratification was used for this study.

We have made these changes in the revised manuscript (page 28, lines 1, lines 3-4, lines 10-11; red text).

Line 334. Change to “Compared to the microbial communities…”

We have made the change. Thanks! (page 15, line 1, red text).

Line 607. Why do you think the descending (culturable) microbes might have a significant role in the hadal sediment microbiome when they were a tiny fraction of the overall community abundance, as reflected in the metagenomes? The authors should quantify the fraction of microbiome represented by the cultured isolates.

We appreciate the comments about the roles of descending microbes in the composition of the hadal sediment community. After going through their numbers (for isolates; see next paragraph below), we came to realize that we were not prudent in the discussion in suggesting "a significant role" of the descending microbes.

Our isolates were found to concentrate to four phyla (Proteobacteria, Bacteroidetes, Actinobacteria, and Firmicutes) out of more than 20 phyla/candidate phyla represented by the MAGs. Furthermore, the three genera (Halomonas, Pseudoalteromonas, and Idiomarina) that represented the top isolates in abundance, accounted for 0.14%, 0.08% and 0.19%, respectively, in the relative sequence abundance of the microbiome. Therefore, we have modified the text in the revised manuscript by removing the suggestion of 'a significant role' (page 25, lines 23-25, red text).

Line 835. Anaerobic agar culturing. Despite the treatment of the agar to remove oxygen, the agar media were inoculated under aerobic conditions in the lab correct? A anaerobic hood was not used correct? This information should be included. And the authors should make it clear that the conditions used would not have enabled the isolation of strict anaerobes, only facultative anaerobes.

Both are correct. The dilution and inoculation were performed under aerobic conditions in the lab. (We did not use anaerobic hood for dilution and inoculation, partly because the sediment samples had been initially exposed to air when retrieved.) Per the suggestion, we have clarified it in the revised manuscript: texts updated in the 'Results and discussion' (page 24, lines 21-22; red text) and the 'Materials and Methods' (page 35, lines 1-2; red text). We appreciate these comments.
